# Multi-Color Spectral Transcript Analysis (SPECTRA) for Phenotypic Characterization of Tumor Cells

**DOI:** 10.3390/biom3010180

**Published:** 2013-02-11

**Authors:** Joanne H. Hsu, Jingly F. Weier, Heinz-Ulrich G. Weier, Yuko Ito

**Affiliations:** 1 Fred Hutchinson Cancer Research Center, 1100 Fairview Avenue North, Seattle, WA 98109, USA;; 2 Life Sciences Division, E.O. Lawrence Berkeley National Laboratory, 1 Cyclotron Road, Berkeley, CA 94720, USA; E-Mail: ugweier@lbl.gov (H.U.G.W.); 3 Department of Pathology, University of California, San Francisco (UCSF), CA 94143, USA; E-Mail: jinglyw@gmail.com (J.F.W.); 4 National Institute of Science and Technology Policy (NISTEP), Ministry of Education, Culture, Sports, Science and Technology, Tokyo 100-0005, Japan; E-Mail: itoh@nistep.go.jp (Y.I.)

**Keywords:** cancer, gene expression, tyrosine kinase, fluorescence *in situ* hybridization (FISH), spectral imaging

## Abstract

Many human tumors show significant changes in their signal transduction pathways and, thus, the way the cells interact with their environment. Often caused by chromosomal rearrangements, including gene amplifications, translocations or deletions, the altered levels of gene expression may provide a tumor-specific signature that can be exploited for diagnostic or therapeutic purposes. We investigated the utility of multiplexed fluorescence *in situ* hybridization (FISH) using non-isotopically labeled cDNA probes detected by Spectral Imaging as a sensitive and rapid procedure to measure tumor-specific gene expression signatures. We used a commercially available system to acquire and analyze multicolor FISH images. Initial investigations used panels of fluorescent calibration standards to evaluate the system. These experiments were followed by hybridization of five-to-six differently labeled cDNA probes, which target the transcripts of tyrosine kinase genes known to be differently expressed in normal cells and tumors of the breast or thyroid gland. The relatively simple, yet efficient, molecular cytogenetic method presented here may find many applications in characterization of solid tumors or disseminated tumor cells. Addressing tumor heterogeneity by means of multi-parameter single cell analyses is expected to enable a wide range of investigations in the areas of tumor stem cells, tumor clonality and disease progression.

## 1. Introduction

The field of molecular cytogenetic technology is comprised of various approaches to determine the chromosomal make-up of diploid cells, spermatocytes or polar bodies in research and clinical practice. While the chromosomal analysis using fluorescence *in situ* hybridization (FISH) has dominated the field for many years, DNA microarray-based assays have gained popularity in recent years due to the fact that they are relatively easy to automate and offer superb resolution. Our laboratory has been adapting DNA microarray technology to identify genes involved in the onset and progression of endocrine tumors in the thyroid, breast and prostate. Not too long ago, the expression levels of only a few genes could be assayed in a single experiment. The cDNA microarrays now circumvent this limitation [1,2]. However, problems related to tumor heterogeneity limit our progress in cancer research. Most tumors represent a mixture of different cell types, due to the normal complexity of the tissue, which includes aneuploidy and somatic genomic variation [3,4], as well as the complexity of tumors as they evolve from benign lesions to the more malignant, metastatic neoplasms [3,5,6,7,8,9,10,11]. If researchers collect microarray data without confronting the problem of tumor or tissue heterogeneity, important correlations (e.g., between the spatial or temporal expression of several genes and the tumor or disease stage) might be missed [2,5,12].

Preliminary studies performed in many laboratories indicated that a majority of solid tumors are heterogeneous in regards to cellular oncogene expression. To be able to assess intercellular variability, these studies were often performed using FISH with touching imprint preparations of tumor cells (so-called ‘touch preps’) and filter-based microscope systems [13,14]. Typically, no more than two or three fluorescently tagged hybridization probes were used per experiment [2]. When more expressed sequences had to be analyzed, additional slides from the same specimens had to be hybridized. 

The quantitative PCR and filter-based RNA FISH approaches reported previously [15,16] may work well for homogeneous cell cultures, but they do not allow study of the correlation between the expression levels of five or more genes, if the cells of interest are relatively rare or genes are expressed at vastly different levels from one cell to another [17].

Spectral imaging, on the other hand, offers superior resolution and additional choices for probe labels than the filter-based microscope imaging systems [18,19,20,21,22,23]. This approach is based on exciting the fluorochromes simultaneously in several different wavelength intervals using a custom designed polychromatic mirror and replacing the fixed wavelength bandpass filters in the microscope’s emission path with a combination of a custom multiband emission filter and an interferometer (Sagnac interferometer) [18,24,25]. Commonly referred to as ‘Fourier spectroscopy’, interferograms for all points in the image plane are generated by dividing the light entering the Sagnac interferometer into two beams that travel paths of slightly different lengths before combining the beams at the interferometer exit [24]. A camera attached to the microscope port records a series of resulting interferograms, while the optical path difference is changed stepwise. Next, a spectral image, *i.e.*, a stack of monochrome images representing the image intensities as a function of the wavelength, are calculated on a personal computer for all points in the image by a straightforward mathematical operation called ‘Fourier transformation’ [23,24].

Spectral imaging systems have been used extensively to study structural and numerical chromosome alterations [25,26,27,28,29,30,31,32,33]. If the objects are physically separated, such as the chromosomes in metaphase spreads, probes labeled with different combinations of fluorescent reporter molecules can be hybridized to uniquely decorate the target chromosomes. Each of the 24 human chromosome types (*i.e.*, the 22 autosomes, X and Y), for example, can then be unambiguously identified by comparing the measured spectrum along each chromosome with a pre-recorded library of reference spectra [26]. This technique, termed ‘spectral karyotyping (SKY)’, has been applied very successfully to identify translocations and marker chromosomes in tumor samples and prenatal diagnosis [26,27,29,30,31,33]. Whole chromosome painting probes labeled with chromosome-specific combinations of up to five reporter molecules are commercially available, thereby greatly facilitating the identification of marker chromosomes and cryptic translocations in the clinical practice [34]. 

The work of Fung *et al*. [28,32] extended the application of spectral imaging (SIm) to the analysis of interphase cell nuclei. Chromosome-specific DNA repeat probes and single copy DNA probes labeled with combinations of six reporter molecules allowed the routine enumeration of 10 different chromosome types in single blastomere cells from human preimplantation embryos [28]. The analysis using such ‘combinatorial labeled probes’ [35], however, becomes much more difficult when hybridization domains overlap spatially [32]. Multicolor FISH (mFISH) and SKY or SIm meet their limitations [36,37,38], especially when it comes to the issues of sensitivity and signal flaring or blending caused by probe co-localization, but we felt the advantages of SIm in regard to fluorochrome identification outweigh the acquisition speed advantages of filter-based mFISH systems [19,39]. 

The short-term goal of the present study was the development and thorough evaluation of an affordable, molecular cytogenetics-driven, microscope-based system to quantitatively assess the expression levels of five or six genes of interest. With a research focus on thyroid and breast neoplasms, we selected a panel of genes known to independently alter their relative levels of RNA expression when normal cells undergo malignant transformation or tumors progress. 

With the measurement of intratumoral heterogeneity in mind, we decided to develop a SIm-based analytical technology platform for single cell gene expression profiling that uses gene-specific cDNA probes labeled with individual, distinguishable reporter molecules. Our multi-color ‘Spectral Transcript Analysis (SPECTRA)’ uses fluorochromes that can be distinguished based on significant differences in their emission spectrum. Spatial overlap, an important issue, since most of the target RNA molecules might be found in the cell’s cytoplasm and ribosomes, is addressed by a technique termed ‘spectral un-mixing’ [22,23,25]. Here, we describe the principle components of our SPECTRA system and demonstrate its application for the semi-quantitative analysis of five-to-six tyrosine kinase (tk) RNA species in breast or thyroid epithelial cells.

## 2. Results and Discussion

### 2.1. System Evaluation Using Fluorescent Beads

The Inspeck^TM^ beads commercially available from Invitrogen (Carlsbad, CA) appeared to be better suited than hybridized tumor cells to analyze instrument functions, because they showed a much lower degree of fading in repeated scans. Particle concentrations were adjusted so that a typical field of view contained about 20–50 objects. Results indicated that the spectral imaging software delivers virtually identical results when repeatedly analyzing the same image.

The reproducibility of analyzing the same field of view was investigated by moving the stage after each recording of eleven spectral images. Briefly, analyzing beads located in the center of the images, we found relative standard deviations ranging from 2%–5%. The higher variation values were attributed to inappropriate lateral positioning or the out-of-focus location of beads.

**Figure 1:**
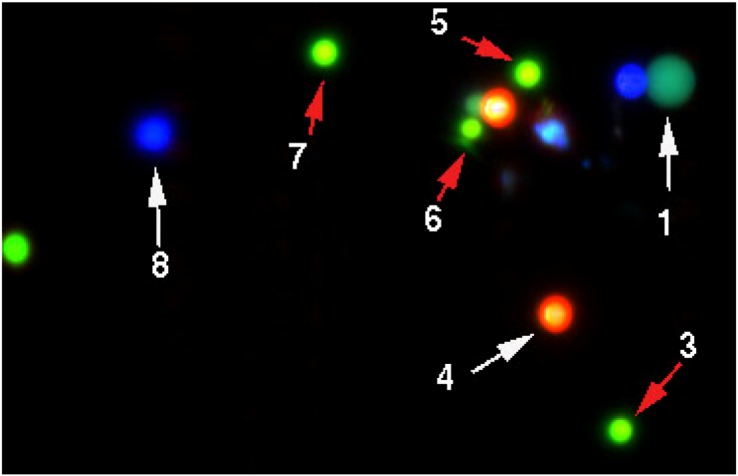
Four-color Inspeck^TM^ beads used to test the reproducibility of the system. The settings for this false-color image display were blue = 475–557 nm, green = 561–635 nm and red = 635–752 nm. The arrows point to non-overlapping signals that were analyzed. Beads 3, 5, 6 and 7 fluoresced in red.

We tested the reproducibility of multi-color fluorescence measurements with fluorescent beads, because they were less affected by photo-bleaching than our hybridized cells. Spectral Images were recorded in a range of 450 nm to 800 nm with a resolution of about 10nm. We prepared slides carrying either five types of beads listed in the Experimental Section or just the four types of Inspeck^TM^ beads (Figure 1), *i.e.*, not using beads with fluorescence emission in the infrared wavelength interval. The reproducibility of measuring the fluorescence spectra of objects was first investigated by recording repeated pictures of the same area without moving the microscope stage and then by moving the microscope stage back-and-forth between the recordings of spectral images. Analysis of beads located in the various parts of the images showed relative standard deviations of 1%–6% for measurements of beads in the 2.5 µm size range, while measurements of smaller (1 µm) beads showed greater variability. The higher variation values were attributed to difficulties in defining the regions of interest and out-of-focus location of beads. As the following results show, average coefficients of variation (CVs) for beads in the relevant size range were well under 4%. Thus, the system possesses the desired reproducibility. 

We prepared mixtures of various types of fluorescent beads or only the four Inspeck^TM^ beads. Typically, 2–3 μL of liquid containing the beads were placed on ethanol cleaned microscope slides and covered with a 22 mm × 22 mm coverslip. Since the slides had a tendency to dry quickly, images were recorded within 24 hours after preparation. When analyzing slides carrying all five types of beads, a typical field of view contained about 50–60 objects. Smaller areas, such as the field shown in Figure 1, were selected for quantitative analysis. 

We prepared a mixture of the four Inspeck^TM^ beads and recorded eleven images. The microscope stage was moved manually between recordings. We analyzed nine of the eleven images, because image AQ1 did not contain all beads and image AQ7 was out of focus. Figure 1 shows a typical false-color image of various beads. The numbers refer to the beads analyzed. 

Table 1 shows the results of our analysis. For each of four beads, Table 1 reports the region-of-interest (ROI) area and average fluorescence per pixel in the relevant spectrum. The column 'total' shows the total amount of fluorescence (area × average intensity per pixel) in the ROI. Table 1 compares the fluorescence emission of four differently colored beads, *i.e.*, beads 1 (orange), 4 (red), 7 (deep red) and 8 (green), in the nine images analyzed. Relative standard deviations range from 2.5% to 6.1% (average 4.05%). The analysis of four red-to-infrared fluorescent beads (beads 3, 5, 6 and 7) (Experimental Section 3.1.) showed less variation. For the red fluorescent beads, relative coefficients of variation ranged from 1.0% to 4.0%, with an average of 2.3% (data not shown).

In summary, the spectral imaging system allowed us to separate complex fluorescence spectra into up to five or six constituents (as shown further below) and to determine the relative contribution of each of the constituents with high reproducibility. Thus, the system meets the specifications required for multiple gene expression profiling.

**Table 1:** Reproducibility of fluorescence measurements using fluorescent beads.

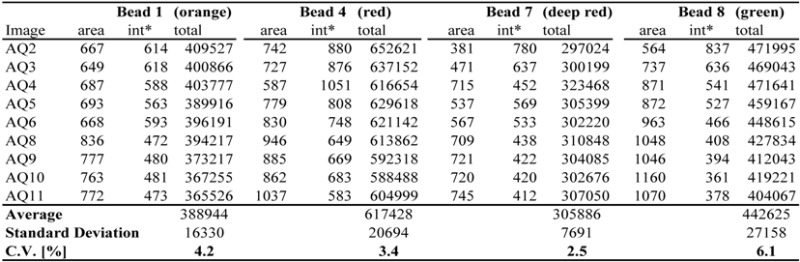

* The 'int' is the average intensity per pixel.

### 2.2. Gene Expression Analysis of Cancer Cell Lines

Key to the success of RNA fluorescence *in situ* hybridization (FISH) is to maintain an essentially RNase-free environment. We achieved this by pretreating all solutions with DEPC, while glassware used in the experiments was pretreated with an RNase inhibitor.

**Figure 2:**
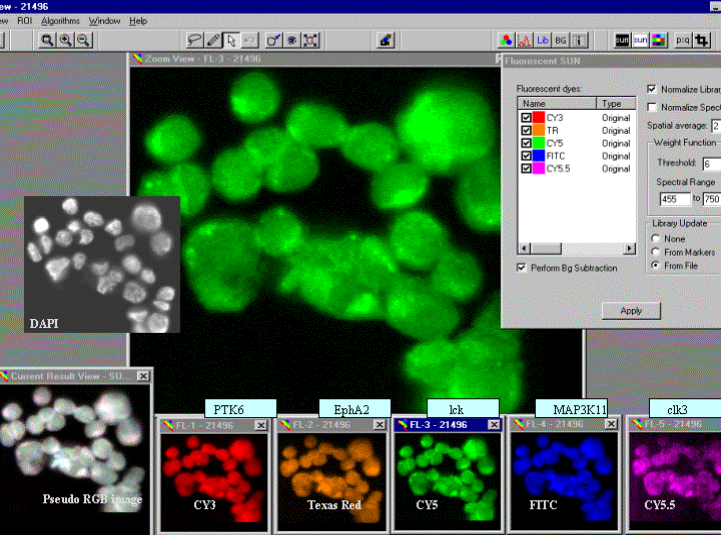
The spectral analysis of MCF-7 cells that were deposited on glass slides and hybridized with the probe mixture listed in Section 3.3. Following background subtraction, the fluorescence spectra emitted from each region-of-interest (ROI) were separated into their five constituents, shown in the five small windows labeled FL-1 to FL-5 at the bottom of the figure. The library files, *i.e.*, prerecorded spectra, used for spectral un-mixing (‘SUN’) are highlighted in the small window in the upper right corner labeled ‘Fluorescent SUN’.

Using the five fluorochromes (FITC, Cy3, Texas Red, Cy5 and Cy5.5) we were able to label, detect and quantitate specific gene transcripts in individual cells. The example in Figure 2 demonstrates the typical spectral analysis of MCF-7 breast cancer cells hybridized with individually labeled cDNA probes against five different tyrosine kinase gene transcripts (homo sapiens PTK6 protein tyrosine kinase 6 (PTK6), ephrin receptor a2 (EFNA2), homo sapiens lymphocyte-specific protein tyrosine kinase (LCK), homo sapiens mitogen-activated protein kinase kinase kinase 11 (MAP3K11) and homo sapiens CDC-like kinase 3 (CLK3)). The deconvoluted or ‘spectrally un-mixed’ images showing the distribution of fluorochromes within cells indicate gene-specific RNA levels and spatial distributions (Figure 2, bottom). For the display, false colors were assigned to the reporter molecule images, as indicated in the box ‘Fluorescent SUN’ in the upper right corner of Figure 2. The image labeled ‘Current Result View’ in the lower left corner shows a pseudo-RGB image of fluorescent signals prior to spectral un-mixing (SUN). A 4’,6-diamidino-2-phenylindole (DAPI) image showing the location of cell nuclei was included in Figure 2 for reference. The next steps of expression analysis involved the definition of regions-of-interest (ROI) and calculation of the amount of fluorescence per dye per cell.

We found that in non-synchronized cell cultures, gene expression levels were affected by the cell cycle position. When measuring the relative amounts of probes bound per cell, however, we were able to discriminate cells from different lines. Future studies will be needed to address the issues of ratio imaging and internal standards. 

We created artificial mixtures of paraformaldehyde (PFA)-fixed MCF-7 (breast cancer) cells and TPC-1 (thyroid cancer) cells that were placed on cleaned glass slides for hybridization. Preliminary studies using poly-L-lysine coated or silanated slides, however, lead to unacceptable levels of background fluorescence. After cell counts were taken with a hemocytometer, we mixed PFA-fixed MCF-7 and TPC-1 cells at ratios of 1:1 and 100:1. In spite of our best efforts to minimize clumping of cells, the cells did stick together to a degree. Therefore, our cell mixture ratios were not precise and, dependant on the field of view, were found to vary by up to a factor of three to four. We opted not to use excessively vigorous measures to break up these clumps to preserve the structural integrity of our cells. Several thousand cells from a given cell mixture were spotted on a glass microscope slide and allowed to dry above a 90 ºC hot plate. The slides did not contact the hot plate, but the additional heat evaporated the water more quickly. Each spot consisting of a mixture of cells was flanked by two spots consisting of each cell type alone. All spots had approximately the same number of cells. The slide-bound cells were then subjected to our hybridization protocol.

**Figure 3:**
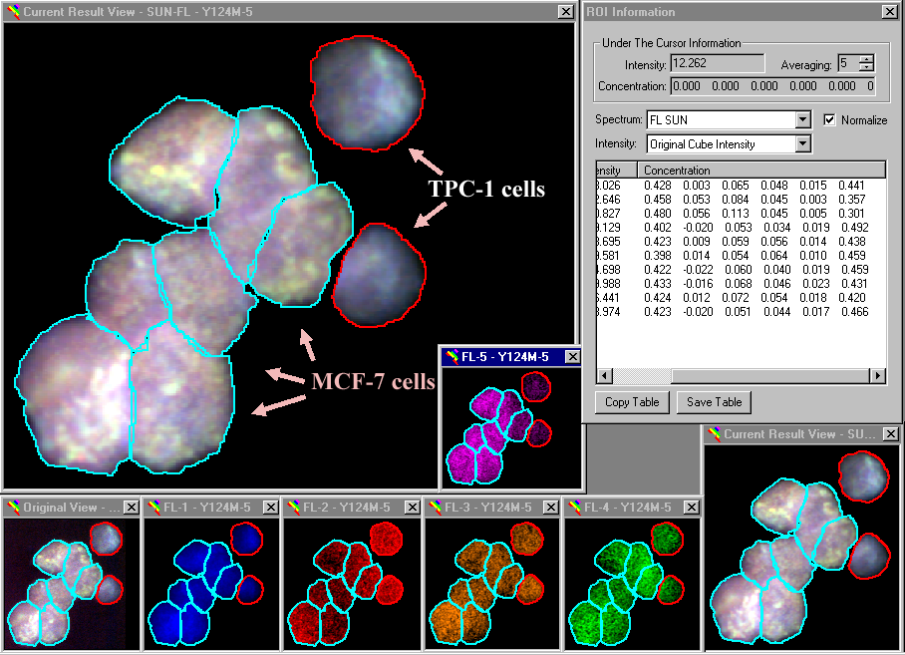
The spectral analysis of mixed cell samples. MCF-7 and TPC-1 cells were mixed, deposited on glass slides and hybridized with the six probe mixture listed in Section 3.3. Following background subtraction, the fluorescence spectra emitted from each ROI were separated into their constituents (shown in Table 2, below). The results are shown in the small window labeled ‘ROI Information’ in the upper right corner. The Figure shows five windows (FL-1 to FL-5) with cDNA fluorescence *in situ* hybridization (FISH) results.

Our analytical procedures included a definition of reference spectra obtained from slides hybridized with just one probe at a time, definition of ROI’s, subtraction of background fluorescence and SUN. A typical example, *i.e.*, the analysis of image Y124M-5, is shown in Figure 3. Our nomenclature is simple: this image depicts the analysis of the 124^th^ slide prepared by Dr. Y. Ito, and we are looking at the fifth image recorded from cells deposited in the middle spot. This slide (Y124) contained one spot each of pure MCF-7 and TPC-1 cells to the left (Y124L) and right (Y124R), respectively, and a 50:50 ratio of these cell lines in the spot in the middle. Spectral analysis showed a cluster of seven MCF-7 cells next to two TPC-1 cells (Figure 3).

The high level of FITC fluorescence emitted from MCF-7 cells stems from the cDNA probe used in these experiments. Our RET/PTC probe was a chimeric cDNA clone isolated in Dr. S. Jhiang’s lab (Ohio State University, Columbus, OH) from a tumor carrying a RET/PTC3 rearrangement [40]. In the rearrangement, the 3’-end of the RET gene containing the entire tyrosine kinase domain is fused to the 5’-end of a constitutively expressed gene (called ‘ELE1’ or ‘RFG’) [10,40]. The FITC fluorescence measured in MCF-7 cells is believed to represent the ELE1 portion of the probe plus any amount of autofluorescence not removed by the background subtraction. To eliminate this cross-reactivity, we plan to isolate only the 3’-end of the RET/PTC3 cDNA clone to be used as a probe in further investigations. 

The breast cancer cells, on the other hand, show higher levels of ABL and PTK6, which comes as no surprise, since the latter was first reported to be expressed at high levels in breast cancer tissues. The analysis of additional images consistently showed that TPC-1 cells expressed higher levels of RET and lower levels of ABL. 

**Table 2:** Relative levels of six fluorescent reporters used to tag RNA species.

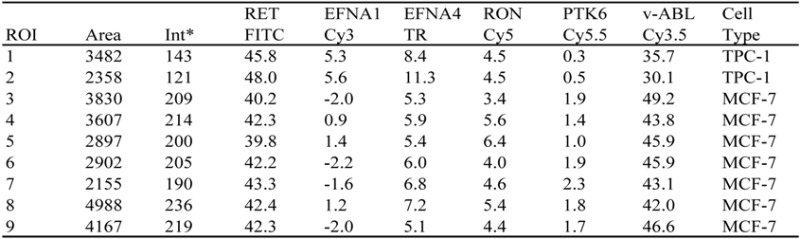

* Fluorescence levels are reported in percent of total cellular fluorescence in image Y124M-5, recorded from a mixture of TPC-1 and MCF-7 cells

### 2.3. Detection of Rare Cells that Differ in Target Gene Expression by 50% or More from the Majority of Cells

We used the above mentioned 1:100 mixture of TPC-1 and MCF-7 cells to investigate the system’s ability to detect rare events. Due to an inversion on chromosome 10q, which leads to expression of a chimeric RET/PCT1 transcript [40], TPC-1 cells are expected to express the RET gene at a much higher level than MCF-7 cells. Following hybridization, we recorded eleven images containing 79 cells. One of the images could not be analyzed, because it was recorded with mismatched filters. Visual inspection of the ten fields of view prior to image acquisition had suggested the possibility that two of them might contain one or two TPC-1 cells, and one image contained a large cluster of green fluorescent cells. Spectral analysis revealed that the clusters of cells in some images were comprised entirely of TPC-1 cells, while other images contained only MCF-7 cells (data not shown). 

In conclusion, the spectral imaging system and digital imaging processing are capable of detecting rare cell that differ in target gene expression by 50% or more from the majority of cells. Artificial mixing experiments using TPC-1 and 184A1TH cells [41] showed similar results (data not shown).

## 3. Experimental Section

### 3.1. Fluorescent Beads

We used fluorescent beads (Inspeck^TM^; Invitrogen) to test the reproducibility of the system. Inspeck^TM^ beads are available in different absorbance/fluorescence wavelength intervals, in different sizes, as well as with different average amounts of dye load. In the experiments described above, we used four different colors (green orange, red, deep red) of either 2.5 μm or 6 μm beads loaded with either 0%, 1%, 3%, 10% or 100% of the respective fluorochrome to evaluate our system. The bead types can easily be distinguished by eye. Table 3 lists the Inspeck^TM^ beads that we used in our experiments and the fluorescence characteristics of 3 types of calibration standard beads that fluoresce in the infra-red wavelength region. All system-specific reference spectra for Inspeck^TM^ beads and infrared dyes were generated from background fluorescence-corrected spectral images of individual beads and saved as library files to make them available for the analysis of additional images.

**Table 3:** Fluorescent beads used to evaluate components of the SPECTRA system.

Color	Type	Size	Excitation	Emission
			[nm]	[nm]
Green	Inspeck^TM^	2.5 micron	505	515
Orange	Inspeck^TM^	2.5 micron	540	560
Red	Inspeck^TM^	2.5 micron	580	605
Deep Red	Inspeck^TM^	2.5 micron	633	660
Crimson	Fluosphere^TM^	1 micron	625	645
Scarlet	Fluosphere^TM^	15 micron	645	680
Ultra Red	Peakflow^TM^	6 micron	665	695

### 3.2. Tissue Specimens

The MCF-7 cells (ATCC, Rockville, MD) and 184A1TH cells [41] were grown in a medium consisting of DME H-16 50%/F-12 50% mix, 10% fetal calf serum, 1% penicillin/­streptomycin (100% is 10,000 μg/mL streptomycin plus 100 units/mL penicillin) and insulin (4 μg/mL at 0.024 units/μg). TCP-1 cells [42] were grown in Minimal Essential Medium (MEM) Alpha medium with L-glutamine (Invitrogen, La Jolla, CA, USA), 5% fetal calf serum (Invitrogen) and 1% penicillin/streptomycin. Prior to fixation, the cells were grown in T75 culture flasks (Corning Inc., Corning, NY, USA) to about 80% confluence and then trypsinized using 0.5 mg/mL trypsin in 0.2 g/L EDTA (Invitrogen), 1.0 g/L glucose, 0.58 g/L NaHCO3 (UCSF Tissue Culture Facility, San Francisco, CA, USA). Clumps were broken up by repeated pipetting. The cells were then pelleted in a clinical centrifuge at 1,000 rpm for 5 minutes, and the supernatant was removed. Cell pellets were resuspended in freshly prepared 4% PFA (weight-to-volume) in phosphate-buffered saline (PBS), pH 7.4, and incubated for 15 minutes at room temperature. Sensitive *in situ* hybridizations of DNA probes to intracellular RNA targets rely on an RNAse-free environment during and after fixation. To eliminate RNAses, all glassware used was treated with RNase Zap (Ambion, Austin, TX, USA), and all solutions were treated with diethylpyrocarbonate (DEPC; Sigma, St. Louis, MO, USA). Having completed a 15 minute incubation in PFA fixative, the cells were pelleted at 1,000 rpm for 5 minutes. The supernatant was then removed, and the cells were washed three times for 15 minutes each with RNAse-free PBS followed by centrifugation and supernatant removal. Finally, cells were resuspended in RNAse-free PBS at a convenient concentration to drop onto glass slides.

### 3.3. Fluorescence *in situ* Hybridization

We developed a five cDNA probe, six-color *in situ* hybridization protocol to semi-quantitatively analyze the intracellular contents of five potentially oncogenic RNA species (Table 4). Commercially available cDNAs (Research Genetics, Huntsville, AL, USA) were labeled by random priming. Table 4 show the labeling scheme and fluorescence characteristics. 

**Table 4:** Six-color, five transcripts spectral analysis.

RNA/DNA Target	Primary label	Secondary label	Excitation	Emission
			[nm]	[nm]
MAP3K11	Biotin	FITC	495	528
PTK6	Cy3	--	552	565
EFNA2	Texas Red	--	596	620
LCK	Cy5	--	650	667
CLK3	Digoxigenin	Cy5.5	678	703
Genomic DNA	DAPI	--	363	461

The five color labeling scheme was designed to match the filters and mirror the spectral imaging system (Applied Spectral Imaging, Ltd. (ASI), Carlsbad, CA, USA). These were filters designed for use in SKY experiments (ChromaTechnology, Bellows Falls, VT, USA) using whole chromosome painting probes (ASI). Probe DNA were isolated from overnight cultures of the cDNA clones 2189231 and 966003 probes (Research Genetics) using the GenElute plasmid miniprep kit (Sigma), according to the manufacturer’s instructions. The inserts of the other three cDNA clones were amplified by PCR using vector-specific primers. PCR conditions were as follows: denaturation at 92 °C for 1 min, primer annealing at 42 °C for 2 min and extension at 72 °C for 3 min for a total of 25 cycles. The PCR products were precipitated in 1.5 volumes of 2-propanol and resuspended in one fifth of the PCR volume. We incorporated one of five different reporter molecules (biotin, Cy3, Texas Red, Cy5 and digoxigenin) into our probe cDNAs by random priming using a commercially available kit (BioPrime^TM^, Invitrogen). The cDNA labeling and detection scheme is shown in Table 4. We combined 0.5 µL of each cDNA probe with 1 µL salmon sperm DNA (20 mg/ml; 3'–5', Boulder, CO) and 1 µL human COT1^TM^ DNA (Invitrogen), before 7 µL of hybridization master mix [(78.6% FA), 14.3% dextran sulfate in 2.9× saline-sodium citrate (SSC) buffer, pH 7.0 (1× SSC is 150 mM NaCl, 15 mM Na citrate))], were added. This gave a total hybridization mixture of 11.5 µL. 

The hybridization mixture was denatured at 91 °C for 5 min, then placed on ice for about 12 min. The slides were denatured on a hot plate for 3 min at 91 °C, then dehydrated in 70%, 80% and 100% ethanol for 2 min each step and allowed to air dry. The hybridization mixture was applied to the slides, covered with a glass coverslip and sealed with rubber cement. The hybridization proceeded at 37 °C for 18 hr in a moisture chamber.

Following hybridization, the slides were washed two times at 43 °C in 50% FA/2× SSC for 15 min each, followed by a 1 min wash in 2× SSC at 20 °C. Next, 80 µL of PNM blocking reagent (5% non-fat dry milk in 0.1 M sodium phosphate, pH 8.0, 0.1% nonidet-P40, 0.1 % sodium azide) were applied to each slide, slides were covered with a plastic coverslip and incubated at 20 °C for 5 min. After removal of the coverslip, 80 µL of detection buffer I (Vial 3, SKY^TM^ kit, ASI) containing anti-dioxin antibodies raised in sheep (which cross-reacts with digoxigenin) (5 µg/mL in 0.1% Tween 20/4× SSC, Roche) were added to each slide. The slides were then incubated at 20 °C for 30 min and washed two times in 2× SSC at 20 °C for 15 min each on a shaking platform. After a 5 min blocking step in PNM as before, 80 µL of PNM buffer containing mouse-anti-sheep antibodies conjugated to Cy5.5 (5 µg/mL, Rockland Immunochemicals, Gilbertsville, PA) and avidin-FITC (20 µg/ml, Vector, Burlingame, CA) were applied to each slide, and slides were incubated at 20 °C for 30 min in the dark. Slides were then washed two times in 2× SSC at room temperature for 15 min each on a shaker. Finally, the slides were mounted in 10 µL of DAPI (Vial 5, SKY^TM^ kit) and coverslipped.

Specifically, for the discrimination of thyroid TPC-1 cancer cells known to express a chimeric form of the ret proto-oncogene [40,42] and MCF-7 breast cancer cells, we prepared a second set of cDNA probes targeting the six tk transcripts listed in Table 5. This probe set uses four fluorochromes coupled directly to the cDNA probes as reporter molecules, as well as biotin and digoxigenin, which were detected with Cy3.5-labeled avidin and Cy5.5-labeled anti-digoxigenin, respectively. Staining conditions have been described previously [43,44,45]. 

The cDNA clones used in the six transcript analysis, their accession numbers and insert sizes are listed in Table 6. These probes target two ephrin receptors (A1: EFNA1; A4: EFNA4) and the transcripts of the tk genes ABL, RON, BRK and RET. All cDNA clones, with the exception of RET, were from the I.M.A.G.E. consortium (‘The Integrated Molecular Analysis of Genomes and their Expression’), Lawrence Livermore National Lab., Livermore, CA, distributed by Research Genetics.

**Table 5:** cDNA targets and fluorochromes used in the seven-color, six RNA target spectral analysis.

Target	Primary label	Secondary label	Excitation [nm]	Emission [nm]
genomic DNA	DAP1	-	363	461
RET	FITC	-	495	528
EFNA1	CY3	-	552	565
c-ABL	biotin	CY3.5	581	596
EFNA4	Texas Red	-	596	620
RON	CY5	-	650	667
PTK6	digoxigenin	CY5.5	678	703

**Table 6:** cDNA clones used in the six-target FISH analysis.

RNA	I.M.A.G.E.	Insert Size	Genbank	Fluorescent
Target	clone ID	[kb]	Accession #	Reporter
EFNA1	320514	0.75	W16661	Cy3
EFNA4	1942138	1.8	XM_002578	Texas Red
c-ABL	1118140	2.2	XM_001717	Biotin*(Cy3.5)
RON	2256805	3.3	NM_002447	Cy5
PTK6	182934	0.6	None	Digoxigenin* (Cy5.5)
RET	(PTC3)	1.95	None	FITC

*Detected via incubation with avidin-Cy3.5 or antibodies against digoxin.

### 3.4. ‘Spectral Un-mixing (SUN)’ of High Resolution Images, Image Segmentation and Fluorescence Integration to Measure Intracellular Levels of up to Six Fluorochromes

#### 3.4.1. Image Acquisition

SIm combines the techniques of fluorescence microscopy, a charge-coupled device (CCD) camera and Fourier spectroscopy. Our system dedicated to image acquisition is comprised of a Zeiss Axioskope microscope (Zeiss, Oberkochen, Germany) equipped with a 150 W xenon light source (Ludl, Novato, CA, USA) and standard 40×, 63× and 100× oil immersion lenses for fluorescence microscopy (Zeiss), an ASI SD200 SpectraCubeTM spectral imaging system interferometer and a Vosskuehler (Osnabrueck, Germany) CCD camera. All images were acquired using the SpectraCube program (ASI). A second computer workstation runs a copy of SpectraView (Version 1.6, ASI) to allow off-line processing of spectral images. Both computers have the SkyView software (ASI) installed, which allows us to perform spectral karyotyping and spectral imaging analyses.

Images were acquired as described [18,28] using the spectral imaging software (ASI). Parameters were set to acquire spectral images from 450–800 nm. The multiple band pass filter set used for fluorochrome excitation was custom-designed (SKY-1, Chroma Technology, Brattleboro, VT, USA) to provide broad emission bands (giving a fractional spectral reading from ~450 nm to ~850 nm). Using a xenon light source, the spectral image was generated by acquiring 80–130 interferometric frames per object. Next, each interferogram was Fourier-transformed, producing the fluorescence spectrum for each pixel of the image. DAPI images were recorded using a DAPI specific optical filter set and stored as ‘.tiff’ files. Sample emission spectra were measured in the visible and near-infrared spectral range simultaneously at all points in the microscopic image. For quality control, the spectral information was displayed by assigning red, green or blue (RGB) pseudo-colors to three areas of interest in the spectrum before the image stacks were archived.

#### 3.4.2. Digital Image Analysis

Digital image analysis using the SkyView software (ASI) (*i.e.*, the spectral classification of physically separated objects) could be done on either of our computer systems. Spectral analysis to determine the relative intracellular amounts of reporter molecules required the SpectraView software (ASI) and is a rather time-consuming process. In order to avoid bottlenecks at the microscope and image acquisition station, this analysis was done offline on a separate computer workstation. For the present studies, reference spectra libraries were built by recording the fluorescence emission spectra of pure dyes as described [26,28,32]. Data management was facilitated through the use of Case Manager software (ASI), which is a databasing program based on Microsoft Access (Microsoft Inc., Seattle, WA, USA). 

The typical analysis procedure involved creation of a case record in which all relevant information, as well as comments, were stored. The spectral image was then loaded and displayed according to the settings of the RGB-display manager. SpectraView allows us to define up to 9 reference spectra plus a background spectrum for analysis. The common steps in the interactive analysis are comprised of definition and subtraction of background fluorescence, definition or import of reference spectra recorded from pure dyes and spectral un-mixing (SUN) to resolve the spectral image into its spectral constituents.

To obtain quantitative information, the user defines one or several regions of interest (ROI). The SpectraView software allows the user to enter an ROI manually or to use a ‘seed’ ROI and have the program find areas of similar intensity and/or spectral composition. The software will then analyze each pixel within the ROI and attempt to deconvolute the measured spectrum to match the reference spectra. Results are reported as ROI area and average pixel intensity, as well as the relative contribution of each of the reference spectra to the total intensity. The present version of the software reports occasional negative values for the relative contribution. These values will be reported as zeros in future releases.

Initially, we used five fluorochromes (FITC, Cy3, Texas Red, Cy5 and Cy5.5) to detect and quantitate gene-specific transcripts in individual cells. The fluorochromes were chosen to match the SKY-1 filter set and were used very successfully in our SKY and spectral imaging studies to label individual chromosomes in interphase or metaphase cells [32]. Genomic DNA was counterstained with DAPI, bringing the total number of colors to six. However, DAPI images had to be acquired using a DAPI-specific filter set (ChromaTechnology). The RNA targets and labels, as well as excitation and emission characteristics, of the fluorochromes are summarized in Table 4 and Table 5. This set-up allowed us to optimize hybridization and staining conditions. We noted that the signals faded during the time it took to record a full spectrum. We thus decided to use fluorescent beads in initial experiments to investigate the system reproducibility. All fluorescent beads were purchased from Molecular Probes (Eugene, OR, USA). We used four kinds of beads from the Inspeck^TM^ series (3% dye load, 2.5 micron) with fluorescence colors described as green orange, red and deep red (Table 3). One of the excitation bands of the SKY-1 filter set (ChromaTechnology) is centered around 640 nm. To achieve our goal of five different fluorescence colors, we added one of the three beads with infrared fluorescence (Crimson, Scarlet, Ultra Red; Molecular Probes; Table 3). The emission characteristics of the beads are listed in Table 3. The largest beads available with ‘Crimson’ fluorescence were 1 μm Fluosphere^®^ beads.

## 4. Conclusions

The analysis of RNA transcript levels by conventional methods [46,47] or using high density cDNA microarrays [2] provides a wealth of information expected to lead to new clues to carcinogenic processes. Because of the complex and heterogeneous nature of most tumor samples, histochemical techniques, particularly RNA fluorescence *in situ* hybridization (FISH), are required to test predictions from cDNA microarray expression experiments.

Preliminary studies performed in our lab and elsewhere indicated that solid tumors are heterogeneous with respect to oncogene expression. To address this problem, we are developing an innovative system to simultaneously measure cell-by-cell levels of multiple tumor markers. Our approach to multi-color analysis of RNA expression levels using high resolution spectral imaging and digital image analysis has proven to possess the sensitivity, specificity and reproducibility required to detect tumor cells with an abnormal pattern of tk gene expression at frequencies as low as one in a hundred cells. This system, capable of deconvoluting images captured from objects stained with probes carrying one of multiple fluorochromes, was initially tested with fluorescent beads. We then used it to analyze the expression levels of five-to-six different tk genes in MCF-7 breast cancer or TPC-1 cells. Results demonstrate that the SIm system is well qualified to resolve signals from different targets, and quite simple image processing algorithms allow tumor cell classification.

It should be noted that the SPECTRA approach and quantitative PCR (qPCR) complement one another. As shown in the examples above, SPECTRA allows a simultaneous measurement of the amounts and intracellular distribution of several protein-coding or noncoding RNA transcripts and reveals their spatial relationships. The qPCR approach to measure transcriptional levels provides average values over the sample sizes. While it appears straightforward to extend the applications of SPECTRA to the multi-transcript analysis of tissue sections, qPCR following tissue microdissection provides rapid means to study the distribution of single gene expression in tissue sections. Compared to qPCR, SPECTRA analysis offers a lower sample throughput and relies on expensive multi-spectral imaging equipment.

## Disclaimer

This document was prepared as an account of work sponsored by the United States Government. While this document is believed to contain correct information, neither the United States Government nor any agency thereof, nor The Regents of the University of California, nor any of their employees, make any warranty, express or implied, or assumes any legal responsibility for the accuracy, completeness or usefulness of any information, apparatus, product or process disclosed, or represent that its use would not infringe privately owned rights. Reference herein to any specific commercial product, process or service by its trade name, trademark, manufacturer or otherwise does not necessarily constitute or imply its endorsement, recommendation or favoring by the United States Government or any agency thereof or The Regents of the University of California. The views and opinions of the authors expressed herein do not necessarily state or reflect those of the United States Government or any agency thereof or The Regents of the University of California.
